# Systematic Review of Policies and Interventions to Prevent Sexual Harassment in the Workplace in Order to Prevent Depression

**DOI:** 10.3390/ijerph192013278

**Published:** 2022-10-14

**Authors:** Francisco Diez-Canseco, Mauricio Toyama, Liliana Hidalgo-Padilla, Victoria J. Bird

**Affiliations:** 1 CRONICAS Center of Excellence in Chronic Diseases, Universidad Peruana Cayetano Heredia, Lima 15074, Peru; 2 Unit for Social and Community Psychiatry, Wolfson Institute of Population Health, Queen Mary University of London, London E13 8SP, UK

**Keywords:** workplace policies, workplace interventions, sexual harassment, depression, systematic review

## Abstract

Background: Sexual harassment in the workplace (SHWP) is highly prevalent and has a negative impact, including depression, on its victims, as well as a negative economic impact resulting from absenteeism and low productivity at work. This paper aims to outline the available evidence regarding the prevention of depressive symptoms among workers through policies and interventions that are effective in preventing SHWP. Methods: We conducted two systematic reviews. The first focused on the association of depression and SHWP, and the second on policies and interventions to prevent SHWP. We conducted a meta-analysis and a narrative synthesis, respectively. We identified 1831 and 6107 articles for the first and second review. After screening, 24 and 16 articles were included, respectively. Results: Meta-analysis results show a prevalence of depression of 26%, as well as a 2.69 increased risk of depression among workers who experience SHWP. Variables such as number of harassment experiences and exposure to harassment from coworkers and other people increase this risk. Conclusions: There is limited evidence regarding the effectiveness of policies and training to prevent SHWP, mostly focused on improvements in workers’ knowledge and attitudes about SHWP. However, there is no available evidence regarding its potential impact on preventing depression.

## 1. Introduction

Over the past 25 years, the issue of sexual harassment in the workplace (SHWP) has become more recognized [1]. Sexual harassment is defined as any unwelcome sexual behavior that is intimidating, hostile or offensive, which violates the victim’s human rights and dignity [2,3,4,5], and can be physical, psychological, verbal and non-verbal [2]. Behaviors that fall under this definition can range from sexual gestures and comments, unwelcome touching or repeated requests for dates to sexual violence and rape [1,2].

SHWP is a very common problem, with a prevalence between 20% and 50% reported in studies conducted in high-income countries (HICs) [2], and from 14.5% to 98.8% in low- and middle-income countries’ surveys (LMICs) [6]. Despite its high prevalence, it is usually underreported due to fear of retaliation or uncertainty about how to report it [2,3].

Most cases of SHWP can be categorized into two types: quid pro quo and hostile work environment [1,2,3]. Quid pro quo involves asking for sexual favors or allowing sexual advances in exchange for a job benefit or the continuation of employment. Rejection will result, explicitly or implicitly, in an action that affects the victim’s work. A hostile working environment is the result of the harassing behaviors towards the worker and, depending on the frequency and severity, evolve into an unbearable situation for the victim.

Certain workers are exposed to risk factors that increase their odds of experiencing SHWP [2]. Some examples include domestic workers, informal workers and people working with the public (patients, customers, etc.) [2,7]. Informal work is particularly relevant in LMICs, since it is more common [1].

SHWP has a significant negative impact on victims’ wellbeing [2,5]. Consequences include feelings of irritation, anger, fear, humiliation and stress [1]. Furthermore, it can result in workers missing career opportunities or resigning from their jobs [1,2,5]. Perhaps one of the most important consequences is depression, which is two to five times more frequent among people who experienced SHWP, compared to those who do not [8]. The psychological consequences of sexual harassment may be related to the type and severity of sexual harassment, the role of the perpetrator (e.g., coworkers, supervisors, clients), the victim’s attributes, and the access to social support and resources, among others.

In turn, depression has a negative impact on the workers’ quality of life [6,9] and a negative economic impact due to absenteeism and low productivity at work [2]. Therefore, addressing and preventing SHWP can, in turn, help reduce the prevalence of depression and its negative consequences among workers.

Both men and women can be victims of sexual harassment; however, women are more commonly affected [2,6,9]. Currently, there are several international policies and standards aimed at eliminating discrimination and violence against women, including SHWP. Examples of the international policies include the International Labour Organization (ILO) Declaration of Philadelphia, the ILO Convention No. 111, the United Nations Convention on the Elimination of All Forms of Discrimination against Women, and the Beijing Declaration and Platform for Action, among others [2,7]. In addition, the United Nations Sustainable Development Goals include, among them, eliminating all forms of violence against women (Goal 5) and creating productive and decent work for all (Goal 8) [2]. These act as frameworks for businesses’ internal policies and initiatives.

Training is a common initiative to prevent SHWP, aiming to inform workers about internal policies, procedures and resources available, improve knowledge and attitudes regarding gender and help initiate change in the organizational culture [2,10]. Currently, an increasing number of workplaces provide training for managers, supervisors and workers, covering topics such as attitudes, stereotypes, social norms, unconscious bias, and bystander intervention, among others [2]. However, there is still limited evidence aiming at assessing the effectiveness of these interventions and its potential implications for the mental health of the workers.

This paper aims to outline the available evidence regarding the prevention of depressive symptoms among workers through policies and interventions that are effective in preventing SHWP. Specifically, we focused on three research questions: (1) What is the association between depression and SHWP? (2) What policies and interventions are effective at preventing SHWP? and (3) Do these policies and interventions have an impact on the prevention of depressive symptoms? In order to answer these questions, we conducted two systematic reviews, one focused on the association of depression and SHWP (RQ 1), and a second focused on policies and interventions to prevent SHWP (RQ 2 and 3).

## 2. Materials and Methods

### 2.1. Search Strategies

We conducted two systematic reviews, a meta-analysis for the first review and a narrative synthesis for both reviews. Both systematic reviews consisted of database searching. The search was conducted on 22 and 30 November on PubMed, PsycINFO, EMBASE, CENTRAL and Global Index Medicus. In addition, for the review focused on policies and interventions to prevent SHWP (review 2), we searched grey literature via the websites of six organizations (UN, UN Women, ILO, World Health Organization, National Institute of Health and Care Excellence, and Institute for the Study of Labor). The search strategy for both reviews can be found in Supplementary Material A. Key terms included “sexual harassment”, “workplace”, “intervention”, “depression”, among others.

We set out inclusion and exclusion criteria a priori for both reviews. Inclusion criteria were (1) published from the year 2000 onwards, (2) published in English or Spanish and (3) focused on working population. The exclusion criteria were (1) publications such as reviews, case reports, editorials, correspondence and conferences, and (2) publications in other languages.

### 2.2. Data Extraction and Analysis

**Figure 1:**
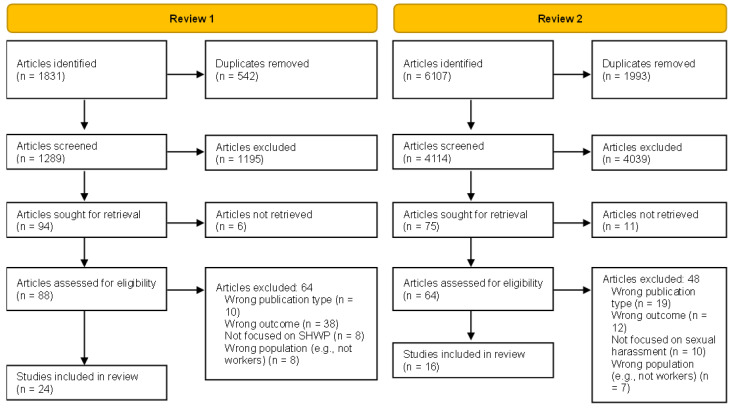
PRISMA diagram.

A minimum of two reviewers screened each article using the Rayyan software [11]. We identified 1831 and 6107 articles for the first and second review. Duplicates were identified automatically using Rayyan, then screened by the reviewers to ensure only identical papers were removed. After removing duplicates, 1289 and 4114 articles were screened by titles and abstracts to include only the papers related to the topic of the reviews. At this stage, the agreement percentage between raters was 95% (k = 0.415) for the first review and 98% (k = 0.403) for the second. Next, the reviewers read each article in full, leading to 24 articles were included for the first review and 16 articles for the second review. The agreement percentage for reviews 1 and 2 were 76% (k = 0.415) and 87% (k = 0.681) for the second. The reviewers were instructed to opt for including an article rather than excluding it when in doubt about their decision. Disagreements between the reviewers were settled by a third reviewer in virtual meetings, where each of the first two reviewers stated their reasoning to either including or excluding an article and then, with the input of the third reviewer, they reached consensus to make a final decision. The inclusion process is detailed in the Preferred Reporting Items for Systematic Reviews and Meta-Analyses (PRISMA) consort diagram (see Figure 1).

Following this process, two reviewers extracted the relevant information (see Supplementary Material A) into an Excel spreadsheet, and any discrepancies in the information extracted were discussed to reach an agreement. This procedure required both reviewers to compare the data extracted and discuss any differences, revise the original data on the paper and reach a final agreement.

For the first review, a meta-analysis was conducted for papers reporting prevalence and/or odds ratios of depression as a result of SHWP, in order to synthesize the results across different studies. For the analysis of prevalence rates, percentages of individuals reporting depression (as measured on a validated clinical scale, or via clinical diagnosis) for those who had experienced SHWP, and associated 95% confidence intervals (calculated using the Wilson’s method which produces asymmetric confidence intervals in studies with low prevalence rates) were entered into STATA Version 17 (StataCorp LP, College Station, TX, USA). Heterogeneity among studies was estimated based on Cochran Q and reported using I^2^ (and 95% confidence interval of the I^2^). As I^2^ > 75% is considered indicative of high heterogeneity, we used a random-effects meta-analysis applying the metan code. For the meta-analysis of odds ratios, where odds ratios were not directly reported in the papers, these were calculated from a 2 × 2 contingency table for the rates of individuals with and without depression and with experience of no experience of SHWP. The resulting odds ratios and confidence intervals were then entered into STATA v17 and the metan command used to conduct a random-effects meta-analysis. The included papers were not enough to be conduct a meta-regression to explore how participant and study characteristics were linked to depression prevalence rates and ORs. Therefore, the findings are summarized narratively.

For the second review, a narrative synthesis was conducted to summarize the findings from the included studies. This process involved identifying common key topics between the included articles, such as the setting in which the study took place, the participants characteristics, the policy or intervention’s characteristics, duration, frequency, the measures used to assess its impact, and the main findings.

### 2.3. Quality Assessment

Included articles were assessed by two researchers using critical appraisal tools to assess the risk of bias. For the first review, we used the Appraisal tool for Cross-Sectional Studies (AXIS tool) [12]. For the second review, we used the Effective Public Health Practice Project Quality Assessment Tool for Quantitative Studies [13]. The researchers compared their assessments, and any disagreements were discussed and solved among them.

### 2.4. Protocol Registration

This systematic review was registered in PROSPERO (Centre for Reviews and Dissemination, University of York; http://www.crd.york.ac.uk/PROSPERO, accessed on 17 December 2021). Registration number—CRD42021291829.

## 3. Results

### 3.1. Review 1: Association between Depression and SHWP

For the first review, two-thirds of the articles included came from HICs (16/24) [8,14,15,16,17,18,19,20,21,22,23,24,25,26,27,28], followed by articles from LMICs (7/24, 3 from upper-middle-income [29,30,31] and 3 from lower-middle-income countries [32,33,34]), and only one article from a low-income country [35]. Lastly, one article used data from different countries, mostly HICs [36]. Regarding the year of publication, two-thirds of the articles were published in the 2010s (16/24) [8,14,15,16,17,18,19,22,23,24,28,30,31,32,34,36], three articles in the 2000s [25,29,35] and five articles in the 2020s [20,21,26,27,33]. Regarding the methodology, most of the studies were cross-sectional (18/24) [14,15,16,17,18,20,21,22,25,28,29,30,31,32,33,34,35,36], while the rest were cohort studies (6/24) [8,19,23,24,26,27].

The great majority (20/24) used a standardized scale to measure depressive symptoms [8,14,15,16,17,18,19,21,22,23,24,25,26,27,28,30,31,34,35,36], while the rest (4/24) relied on self-reported experience of depression [20,29,32,33]. Further details of the studies can be found in Supplementary Material B.

**Figure 2:**
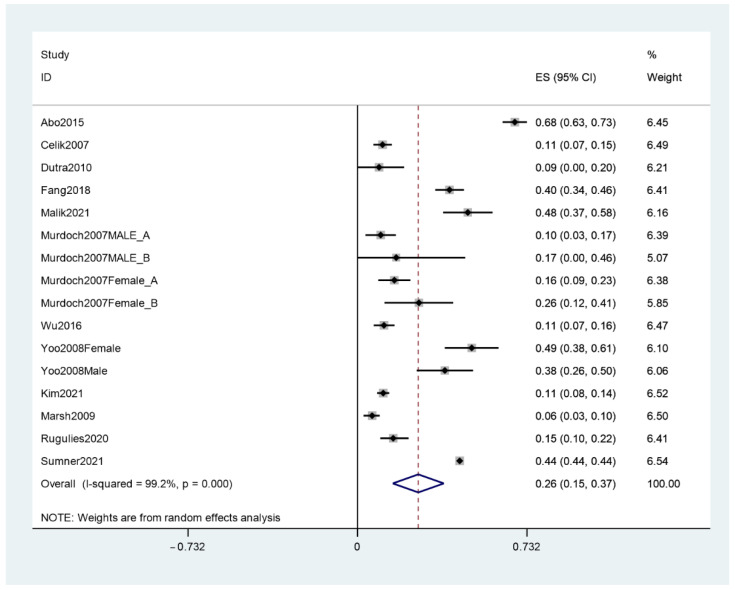
Meta-analysis of the prevalence of depression among workers who experienced SHWP.

Among the included articles, 12 reported the prevalence rates of depression among workers who experienced SHWP [14,20,25,26,27,28,29,30,32,33,35,36]. Meta-analysis results show a combined prevalence of depression of 26% (95% CI, 0.15–0.37). However, the heterogeneity of studies is 99.2% (see Figure 2).

**Figure 3:**
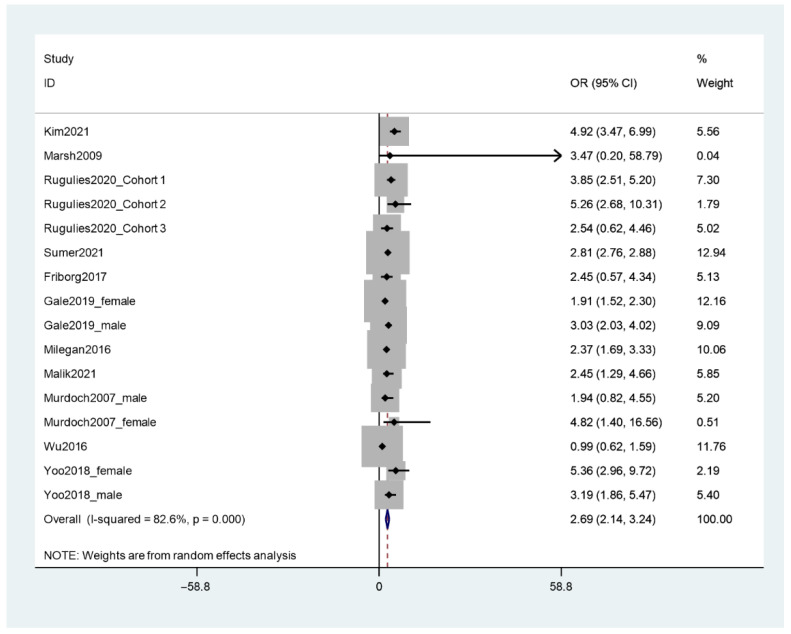
Meta-analysis of odd ratios of depression among workers who experienced SHWP.

Similarly, eleven included articles reported odd ratios of depression as a result of SHWP. The results indicated that workers who experience SHWP have 2.69 higher odds of depression compared to workers who do not experience SHWP (see Figure 3); however, again, heterogeneity was high (82%).

Results from the narrative summary indicate a range of prevalence rates for depression in people who experience SHWP from 5.1% to 67.9%. Similarly, the odds of developing depression following SHWP increase based on the number of SHWP events experienced or who was the perpetrator (see Table 1).

### 3.2. Review 2: Policies and Interventions to Prevent SHWP and Depression

For the second review, most included articles were from HICs, particularly the United States (15/16) [38,39,40,41,42,43,44,45,46,47,48,49,50,51], and one article was from Nigeria, a lower-middle-income country [52]. Around two thirds of the articles were published in the 2010s (10/16) [39,40,41,43,45,46,47,49,50,53], 4 articles in the 2000s [38,42,44,52] and 2 articles in the 2020s [48,51]. Most studies used a cross-sectional design (9/16) [38,39,41,42,46,48,49,50,53], four studies were quasi-experimental [40,43,51,52] and three were RCTs [44,45,47].

Regarding the type of participants, five studies focused on health professionals [43,48,49,50,51], four focused on managers and human resource workers [39,41,45,46], followed by studies focused on the military [42], police [53], government employees [38], university employees [47], and apprentices [52], and two studies on workers from a variety of industries [40,44]. Further details of the studies can be found on Supplementary Material C.

None of the included articles focused on how the prevention of SHWP is associated with the prevention of depression directly. Only one study measured depressive symptoms and burnout. The study controlled for these variables to assess the effectiveness of a computer-based training (CBT) to a CBT plus peer facilitation training [43].

The studies have been categorized into two groups: evidence from policies and evidence from SHWP training.

### 3.3. Evidence from Policies

In total, five articles focused on SHWP policies. One study focused on grievance procedures for SHWP cases and found these are associated with diminished access of women to management positions in settings where men hold more positions of power, and this effect disappears in spaces where women hold more management jobs [41].

The other four studies focused on the impact of SHWP policies on reporting. One experimental study highlighted the importance of having comprehensive and explicit policies. The results showed that a zero-tolerance policy increases the likelihood of reporting SHWP, compared to a less specific policy or no policy [45]. The effect of the policy on the likelihood of reporting the case was higher for a moderate form of SHWP presented (i.e., comments about the body), compared to a more severe quid pro quo SHWP scenario, which is important, since the former is a more common form of SHWP and usually goes unreported.

Another study, focusing on the Dutch police force, showed SHWP rates were slightly higher among police divisions with more comprehensive policies (written statement, grievance procedures, training, etc.), compared to divisions with less comprehensive policies [53]. The authors interpret this result as comprehensive policies increasing awareness of SHWP, leading to more reported cases. Similarly, the other two studies reported an increase in reported cases after implementing the SHWP policy in their workplaces [49,50].

Some of the contents reported in the policies mainly include prevention efforts, reporting and grievance, as well as a clear and explicit stance on SHWP (see Table 2).

### 3.4. Evidence from Training

Eleven included articles focused on training, which can be further subcategorized into two types: observational (4/11) and implementation studies (7/11). Observational studies focused on assessing the impact of SHWP prevention training already taking place in specific work settings, while implementation studies focused on conducting and assessing the impact of training.

Regarding the observational studies, two articles focused on the impact of training on the participants’ perceptions regarding SHWP. One study targeted civil government employees [38], while the other centered around the military [42]. The results from both studies showed benefits from training, including increased self-reported sensitivity to the issue [38], increased awareness of conducts that can be labelled as SHWP, particularly in men receiving the training [38], as well as higher levels of intolerance towards harassment and more perceived efforts from the workplace to prevent it [42].

The other two observational studies focused on specific training features and their impact on preventing SHWP. The first study showed the quantity of training and the interaction between quantity and recency are only modest predictors of increased sensitivity to identify SHWP [39]. The second study revealed that the number of pre-training and post-training activities, such as needs assessment and refresher sessions, respectively, is associated with the perceived success of the training. However, this association is significant only when the perceived reason to conduct the training was related to improving the workplace, instead of legal compliance [46]. In addition, a greater number of post-training activities was associated with a lower perceived frequency of SHWP reports [46].

The implementation studies showed SHWP prevention training has an impact on specific outcomes. The most common and supported evidence was increased knowledge and awareness about SHWP [40,47,48,52,54]. Additionally, in studies where the training aimed to provide specific skills to manage SHWP scenarios, there is a reported increase in confidence [54], self-efficacy [48] and preparedness among workers [51]. One study also identified a decrease in perceived barriers and increased intentions to intervene in these situations [48]. Only two studies measured effects over time in the prevalence of SHWP, and both found a decline in reported harassment at follow-up (6 months) [52,54]. Lastly, one study found training reduced the intention to confront the perpetrator [44]. The researchers attribute this unexpected result to the inclusion of potential consequences of confronting in the training contents, including retaliation and isolation from coworkers, which may have discouraged the participants.

Regarding training methods, nine out of the eleven studies reported in-person training, while the other two compared computer-based with in-person training, finding no significant differences between methods [47,54]. Training usually consisted of a single session lasting around 1 to 1.5 h, mostly using a lecture format with some interactive components, most frequently discussions. Common topics covered were the definition and types of SHWP, as well as how to identify SHWP, with a few studies adding some strategies to manage and address the situation (see Table 3).

### 3.5. Quality of Studies

The studies included in the first review were assessed using the AXIS tool [12]. Overall, the studies included meet the reporting requirements expressed in the tool. However, there were common specific limitations in study reporting, specifically regarding the justification of sample size, information about the non-responders, and the internal consistency of the results (see Supplementary Material D).

Regarding the second review, the studies were assessed using the EPHPP tool. The majority of studies were rated as weak on quality, which is expected considering most were not RCTs, with only two obtaining a moderate score (see Supplementary Material E).

## 4. Discussion

### 4.1. Prevention of Sexual Harassment and Depression

The aim of this review was to examine whether SHWP prevention has an impact on preventing depression among workers. Our first review confirms SHWP is a predictor and a risk factor for depression. In addition, certain variables, such as the number of SHWP experiences and combined exposure to harassment from clients or customers and coworkers, increase the risk for depression. However, we found no direct evidence of the effectiveness of SHWP policies and training on preventing depression. Based on the identified association between SHWP and depression, we can infer early prevention of SHWP, through policies and training, could reduce the risk and prevalence of depression among workers and lead to an improvement in their mental health.

The available evidence regarding SHWP prevention policies and interventions is limited and weak. The most common evidence focused on SHWP prevention training; however, evidence of effectiveness was still somewhat small (11 studies). This result is in line with a report from UN Women conducted in 2020, which states there is a limited amount of literature on training [10]. Despite this limitation, the majority of studies report that both policies and training improve awareness regarding SHWP; and, in some cases, skills to address the situation. However, further research is required to assess the potential effect of training and prevention policies on workers’ depression levels.

### 4.2. Scalability, Applicability and Implementation

The majority of studies included in this review were conducted in formal work environments within HICs, therefore the applicability of our results to other contexts should be carefully assessed and adapted. Studies most commonly focused on health professionals and the military, therefore the results can be easily extrapolated to these workplaces. Further research is required to assess the applicability of these initiatives to LMICs, where informal work is more common. However, most countries adhere to international regulations and have national legislations regarding SHWP [55], which provide the framework to implement SHWP prevention measures.

Implementing SHWP zero-tolerance policies and training can be easily adapted to different types of workplaces with certain policy aspects potentially increasing their impact. Firstly, the workers’ perception of the employer’s intention to implement these initiatives is highly relevant to engage them with the policy [46]. It can be quite common for these policies and training to be implemented only to comply with a legal requirement, or as merely symbolic [10], which can diminish effectiveness. Secondly, it is important to tailor these initiatives to the context in which they are implemented, particularly training, using examples relevant to the types of SHWP situations they may experience and providing relevant information to address and manage it through role-playing [40,43,48,51]. Thirdly, training should be continuous and not restricted to a single moment in time [10]. Fourthly, as identified in the evidence, computer-based and instructor-led training methods provide similar outcomes [47,54]. Therefore, both methodologies can be used in combination or separately, according to each workplace’s conditions and after assessing the skills of the participants, including their technology literacy. Finally, making policies comprehensive and explicit regarding the stance on SHWP will provide a better framework for the workers to report cases [45,53]. Consequently, a key aspect to consider is to develop and inform workers about the grievance procedures that take place after a case is reported. Considering an increased awareness of SHWP could lead to a higher number of cases, as reported in the evidence from policies [45,49,50,53], it is crucial for workers to know how the workplace handles the reports and how they bring them to a resolution.

### 4.3. Strengths and Limitations

To our knowledge this is the first review aiming to outline the available evidence regarding the prevention of depressive symptoms among workers through policies and interventions that are effective in preventing SHWP. We included evidence from both HICs and LMICs.

One limitation is that the great majority of studies identified come from HICs. While it can be inferred that the results would be similar in similar HIC settings, more research is required, particularly for more challenging working conditions such as informal work, which is common in LMICs, and where these results might not be as generalized as for HICs. A second limitation is the concise descriptions of the training contents and methodologies, which made comparison among studies difficult. Given the nature of this review, we did not seek to identify differences in the methodologies used; however, more detailed descriptions of the training contents would have been an important complement to the evidence, as well as a facilitator to replicate the methodologies used. Another limitation is that most studies lack standardized measures to assess the effectiveness of the training programs, mainly relying on self-report of knowledge and attitudes instead of the reported cases of sexual harassment. This also limits the comparability among studies and highlights a gap that needs to be filled in order to better assess and compare the quality of the studies. Other limitations related to the methodology are including only studies in English and Spanish, and from the year 2000 onwards.

## 5. Conclusions

SHWP is a prevalent and important issue to tackle due to its impact on the workers’ lives, particularly their mental health. Workers who experience SHWP are at a higher risk for depression, and certain variables, such as number of harassment experiences and exposure to harassment from people other than coworkers, increase this risk.

There is limited evidence regarding the effectiveness of policies and training to prevent SHWP, and there is no evidence regarding its potential impact on preventing depression. In addition, most of the evidence comes from HICs. Despite this, the evidence indicates that training and policies can improve awareness, knowledge and resources to address SHWP in the daily lives of workers. Due to the aforementioned link between SHWP and depression, it can be inferred that preventing the former would most likely reduce the risk for depression among workers, however further research is required. Regardless of the limited evidence, it is crucial to ensure workplaces set up and implement procedures to address SHWP for the benefit of all workers.

## Figures and Tables

**Table 1:** Narrative summary of the association between depression and SHWP.

Type of Worker	Number of Articles	Main Findings
Health providers	7	Prevalence of depression ranging from 10.8% to 67.9% among those who experienced SHWP [28,29,30,32,33]. On average, workers who experience SHWP score, on average, 9 points higher on depression using the COPSOQ II [17]. SHWP contributes 65.43% in developing depression, anxiety and stress in nurses [34].
Military	6	Prevalence of depression ranging from 5.1% to 44.1% among those who experienced SHWP [14,25,27]. Significantly higher odds of experiencing depression (2.37 and 2.81) among those who experienced SHWP [24,27]. SHWP is a predictor of depression, even after accounting for confounders such as gender, pre-existing symptoms and prior stressors [16,23].
Various workers (more than one type of worker)	5	Higher odds of experiencing depression among those exposed to SHWP [15,20,37]. ORs range from 1.92 to 2.05 if they are harassed by non-workplace personnel (e.g., clients) and 2.45 to 5.26 if they are harassed by coworkers or supervisors. Higher average scores of depressive symptoms in both men and women who experienced SHWP (2.39 and 3.81 points on the GHQ-28) compared to workers who have not been harassed (0.88 and 0.82) [22]. SHWP in the most recent year is associated with depression, even after accounting for previous depression episodes [19].
Airline pilots	1	Overall prevalence of depression of 13.6% among victims of SHWP; however, it ranges from 11.4% if the harassment was experienced only once to 36.4% if experienced four or more times [36].
Cabin crew	1	Workers who experienced one event of SHWP had 1.44 odds of experiencing depression, while those who experienced four or more events had 4.12 odds [8].
Female academic and administrative staff	1	Workers who experienced SHWP had 3.47 higher odds of reporting depression [35].
Female firefighters	1	Higher scores of depressive symptoms on the CES-D among women who experienced sexual harassment at work (20.60, SD = 16.85), in comparison to those without this history (13.46, SD = 13.32) [18].
Fitness instructors	1	Higher scores of depressive symptoms on the BDI among workers who experienced sexual harassment at their jobs, in comparison to those without this history (Z = −2.4, *p* = 0.018) [21].
Hospitality workers	1	SHWP is positively associated with depression (r = 0.24) [31].

**Table 2:** Contents of SHWP policies.

Article	Policy Contents
De Haas [53]	- Grievance procedures to report SHWP and other forms of harassment - Instate confidential advisors to support victims and inform personnel about prevention policies - Increase in number of female police officers to balance the male–female ratio - Inform managers and employees about policies and procedures
Dobbin [41]	By 2002, 98% of employers had grievance procedures, 82% had training for managers, and 64% had training for employees.
Jacobson [45]	Zero-tolerance statement was associated with higher likelihood of reporting SHWP. The statement mentions who are responsible for developing the policy, what it entails and where it is applicable.
Ridenour [49]	Develop violence prevention policies within healthcare Create reporting systems for violent events, and violence prevention committees Write violence prevention plans, violence risk assessments, and post incident response Conduct violence prevention training
Shapiro [50]	Policy includes key programs, such as a professionalism initiative (including a professionalism training session), a disclosure and apology process, peer and defendant support programs, and wellness programs.

**Table 3:** Characteristics of SHWP prevention training (Only eight out of the eleven studies focused on training reported the number of sessions, duration and format).

Article	N° of Sessions	Duration	Topics	Format
Campbell [40]	1	Not reported	- Definition of harassment - Identification/risk reduction - Misconceptions - Prevention	Lecture and discussion with Q and A
Fawole [52]	2	Not reported	- Definition - Types of violence against women - Risk factors - Consequences - Economic rights of young apprentices - HIV/AIDS prevention - How to set up and manage a small business	Lecture with group discussions, Q and A, and case scenarios.
Glass [43]	1	Computer only: 127 min. Computer + peer group training: 3 h approximately	- Definitions - Prevalence of workplace violence and harassment - Strategies (assertiveness, preparedness, establishing boundaries). - Warning signs for harassment - Prevention skills - De-escalation techniques Peer group training: All topics with additional exercises (calming, assertive speaking, body language, and role playing)	Computer-based Peer group: interactive exercises
Goldberg [44]	1	2 h	- Relevant legislation and court decisions regarding SHWP. - SHWP terminology. - Organizational implications of SHWP (policies, grievance procedures, etc.) - Victim responses to SHWP and its ramifications (e.g., retaliation)	Lecture format
Hock [51]	1	50 min	- Prevalence of SHWP - Impact on mental health, work performance and patient care - Preparedness to address it in real time	Lecture with role-playing and script rehearsal to respond to SHWP.
Preusser [47]	1	1.5 to 2 h	- Definition of SHWP - How to handle SHWP	Computer-based or in-person training. In-person training used a lecture format.
Relyea [48]	1	45 to 60 min	- Definition of SHWP - Statistics - Examples and consequences - Intervention strategies (saying something, providing a distraction, delegating someone to intervene or delaying a response until after the incident)	Presentation and group discussions
Shapiro [50]	1	1.5 h	- Discussions on SHWP, bullying, and responses to work-hour requirements - Specific strategies to manage conflict and giving feedback to colleagues who have behaved unprofessionally - Description of institution’s program for addressing concerns	Interactive educational session

## Data Availability

Not applicable.

## Supplementary Materials

The following supporting information can be downloaded at: https://www.mdpi.com/article/10.3390/ijerph192013278/s1, Supplementary Material A: Search terms for databases and data extracted. Supplementary Material B: Included articles in review 1. Supplementary Material C: Included articles in review 2. Supplementary Material D: Quality assessment of studies in review 1. Supplementary Material E: Quality assessment of studies in review 2.
