# Supplementary material for: Systematic Review of Policies and Interventions to Prevent Sexual Harassment in the Workplace in Order to Prevent Depression
Source: Int J Environ Res Public Health. 2022 Oct 14;19(20):13278. doi: 10.3390/ijerph192013278 (PMC9603480; doi:10.3390/ijerph192013278)
Supplement: Supplementary file 1 [file ijerph-19-13278-s001.zip › Supplementary Material A.pdf]

Supplementary Material A. Search terms for databases and data extracted

Review 1. Association of depression and sexual harassment in the workplace

Topic	Outcome
Sexual harassment<br>Workplace harassment<br>Unprofessional behavior	Mental health<br>Depression<br>Depressive disorder

Search string used:

("Sexual harassment" OR "workplace harassment" OR "unprofessional behavior") AND ("mental health" OR depression OR "depressive disorder)

Results:

Database	Search date	Result
Pubmed	22/11/2021	373
PsycInfo	30/11/2021	724
EMBASE	30/11/2021	671
Global Index Medicus	22/11/2021	52
CENTRAL	22/11/2021	11

Data extracted:

- General information: country, author(s), publication year, title and aim of the study
- Methodology: study design, setting, inclusion and exclusion criteria
- Participants' characteristics: age range, mean age, gender distribution, participants' occupation
- Results: depression outcome measured, measure tools used, time window of outcome assessment, main findings

Review 2. Policies and interventions to prevent sexual harassment in the workplace

Topic	Setting	Intervention	Outcome
Sexual harassment<br>Workplace harassment<br>Unprofessional behavior	Workplace<br>Work	Prevention<br>Intervention<br>Policy<br>Practice<br>Strategy<br>Training<br>Management	Mental health<br>Depression<br>Depressive disorder

Search strings used:

(“Sexual harassment” OR “workplace harassment” OR “unprofessional behavior”) AND (workplace OR work) AND (prevention OR intervention OR policy OR practice OR strategy OR training OR management) AND (“mental health” OR depression OR “depressive disorder”)

(“Sexual harassment” OR “workplace harassment” OR “unprofessional behavior”) AND (workplace OR work) AND (prevention OR intervention OR policy OR practice OR strategy OR training OR management)

Results:

Database	Search date	Result
Pubmed	22/11/2021	795
PsycInfo	30/11/2021	2546
EMBASE	30/11/2021	2553
Global Index Medicus	22/11/2021	153
CENTRAL	22/11/2021	60

Data extracted:

- General information: country, author(s), publication year, title and aim of the study
- Methodology: study design, sampling method, setting, inclusion and exclusion criteria, description of policy or intervention, duration and frequency of intervention
- Participants’ characteristics: age range, mean age, gender distribution, participants’ occupation
- Results: outcomes measured, main findings
