# Supplementary material for: Systematic Review of Policies and Interventions to Prevent Sexual Harassment in the Workplace in Order to Prevent Depression
Source: Int J Environ Res Public Health. 2022 Oct 14;19(20):13278. doi: 10.3390/ijerph192013278 (PMC9603480; doi:10.3390/ijerph192013278)
Supplement: Supplementary file 1 [file ijerph-19-13278-s001.zip › Supplementary Material B.pdf]

Supplementary Material B. Included articles in review 1

Author(s)	Publication year	Country	Study design	Population	Sample size	Depression outcome	Results
Abo et al.	2015	Egypt	Cross-sectional	Health professionals<br><br>Nurses	430<br><br>Gender distribution not specified	Self-reported experience	67.9% consider they had depression.
Celik Y, Celik SS	2007	Turkey	Cross-sectional	Health professionals<br><br>Nurses	622<br><br>Gender distribution not specified	Self-reported experience	10.8% consider they had depression.
Dutra et al.	2010	United States	Cross-sectional	Military	54<br><br>100% women	Center for Epidemiological Studies–Depression scale (CES-D)	9.3% of the sample endorsed feeling down, depressed, or hopeless (item score M = 1.72, SD = 0.74), and 17.4% of the sample endorsed anhedonia (item score M = 1.76, SD = 0.85) “at least a moderate amount of the time.”
Fang et al.	2018	China	Cross-sectional	Health professionals<br><br>Otorhinolaryngology physician and nurses	652<br><br>63.7% women and 36.3% men	Chinese version of the Zung Self-Rating Depression Scale (SDS)	39.68% of those who experience non-physical violence in the previous 12 months (verbal abuse, threatening and sexual harassment) present depressive symptoms

Friborg et al.	2017	Denmark	Cross-sectional	Various<br><br>Care work (28.8%), knowledge work (24.9%), industrial work (17.9%), private service (16.3%), building and construction (12.0%)	7063<br><br>54.1% women and 45.9% men	Major Depression Inventory (MDI)	Compared to employees not exposed to sexual harassment, the mean level of depressive symptoms was 2.05 (95% CI: 0.98–3.12) (p = 0.0002) points higher for employees harassed by clients/customers. Employees harassed by a supervisor / colleague / subordinate had a further elevated mean level of 2.45 (95% CI: 0.57–4.34) (p = 0.011) points.
Gale et al.	2019	United States / Canada	Cohort	Cabin crew	4459<br><br>79.4% women and 20.6% men	Patient Health Questionnaire (PHQ)-9	For sexual harassment, the corresponding effect estimates among females were OR = 1.91 for depression (95% CI: 1.52–2.30), among male were OR = 3.03 (95% CI: 2.03–4.02)<br><br>ORs between sexual harassment and depression were 1.44 (95% CI: 0.93–1.95) among those experiencing one event, 1.83 (95% CI: 1.29–2.38) among those experiencing 2–3 events, and 4.12 (95% CI: 3.18– 5.06) among those experiencing 4 or more events.
Gross et al.	2019	United States	Cross-sectional	Military	810<br><br>41.6% women and 58.4% men	Patient Health Questionnaire Depression Scale-8	Deployment sexual trauma (including harassment and assault) was a significant predictor of depression, even after controlling for potential confounders

Hanson et al.	2015	United States	Cross-sectional	Health professionals<br><br>Homecare workers	1214<br><br>100% women	COPSOQ II	Participants who experienced sexual harassment scored, on average 9.0 points higher on depression (p < .001)
Hom et al.	2017	United States	Cross-sectional	Firefighters	290<br><br>100% women	The 20-item Center for Epidemiologic Studies Depression Scale-Revised	Participants with a history of sexual harassment while on the job as a firefighter reported significantly higher levels of CESD-R depression symptoms ($F[1,275] = 11.965$, $p = 0.001$, $\eta 2 = 0.042$), than those without a history of sexual harassment on the job [without = 13.46 (13.32), with = 20.60 (16.85)]
Houle et al.	2011	United States	Cohort	Various	732<br><br>58% women and 42% men	"General Wellbeing Scale" of the Current Health Insurance Study Mental Health Battery	Sexual harassment in the most recent year is positively associated with adult depressive affect, even after controlling for prior depressive affect and other controls.<br><br>Prior harassment at age 29–30 and age 19–26 likewise have statistically significant independent effects on depressive symptoms, net of earlier depressed mood and controls<br><br>No difference between men and women.

Kim HR	2021	South Korea	Cross-sectional	Various<br><br>Respondents of the Korean Working Conditions Survey	50205<br><br>57.1% women and 42.9% men	Self-reported experience	A significant number of the study population had mental health problems associated with violence experienced at work: 2.4% had depressive symptoms<br><br>Compared to those workers who did not experience WPV, workers who experienced sexual harassment were more likely to report depression, adjusted odds ratio (aOR) = 4.92, 95% CI = 3.47-6.99
Malik et al.	2021	Pakistan	Cross-sectional	Health professionals<br><br>Surgeons	146<br><br>100% women	Self-reported experience	47.6% of participants who experienced harassment reported feeling severely depressed vs. 37.1% who have not experienced harassment.
Marsh et al.	2009	Ethiopia	Cross-sectional	Academic and administrative staff members from colleges	387<br><br>100% women	Patient Health Questionnaire (PHQ-9)	6.3% prevalence of depression<br><br>Compared with women reporting no experience of sexual harassment, those who reported sexual harassment had a 3.47 risk of depression (OR=3.47, 95% CI: 0.20-58.79).
Mathisen et al.	2021	Norway	Cross-sectional	Fitness instructors	270<br><br>78.1% women and 21.9% men	Beck Depression Inventory, Version 1a (BDI-1a)<br>Hopkins Symptom Check List (SCL-10) - Depression and anxiety	Higher scores in BDI and SCL-10 among women having experienced verbal SHWP compared to women with no such experiences ($Z = -2.4$, $p = 0.018$; $Z = -3.7$, $p < 0.001$; and $Z = -3.3$, $p = 0.001$, respectively).

							More female group instructors who experienced verbal SHWP scored higher in SCL-10 compared to female GIs with no such experiences (Z = −3.4, p = 0.001; and Z = −2.5, p = 0.01).<br><br>No other statistically significant differences according to sex or profession were found in symptoms of depression.
Matud Aznar et al.	2013	Spain	Cross-sectional	Various	209<br><br>52.6% women and 47.4% men	General Health Questionnaire (GHQ-28)	Men and women victims of SHWP score higher depressive symptoms compared to those who are not victims (men: 2,39 SD=3,67 vs. 0,88 SD=1,63; women: 3,81 SD=4,26 vs. 0,82 SD=1,82)
McCallum et al.	2015	United States	Cohort	Military<br><br>National Guard soldiers	528<br><br>88% men and 12% women	Beck Depression Inventory-II	Reports of sexual harassment during deployment was associated with greater depression symptoms (p = .016).
Millegan et al.	2016	United States	Cohort	Military	37711<br><br>100% men	Patient Health Questionnaire 8-item (PHQ-8)	Men who reported sexual trauma (either harassment or assault) within the previous 3 years had significantly higher odds of depression AOR = 2.37, 95% CI [1.69, 3.33] (over the past 4 weeks) compared to those who did not report sexual trauma.

Murdoch et al.	2007	United States	Cross-sectional	Military	815<br><br>59.8% men and 40.2% women	RAND Mental Health Battery (Short Form)	Both men and women who reported more types of military sexual stressors had significantly more severe depression symptoms than did those who reported fewer or no sexual stressor types<br><br>Men: 6.1% of depression when no experience of SHWP, compared to a range of 8.7% to 16.7% depending on the number of sexual stressors.<br><br>Women: 4.7% of depression when no experience of SHWP, compared to a range of 5.2% to 26.5% depending on the number of sexual stressors.
Mustaq et al.	2015	Pakistan	Cross-sectional	Health professionals<br><br>Nurses	200<br><br>100% women	Depression, Anxiety and Stress Scale (DASS)	Significant correlation of depression with gender harassment, unwanted sexual attention, sexual coercion with r = 0.79, 0.76, 0.72 respectively<br><br>Sexual harassment is contributing 65.43% in developing depression, anxiety and stress among nurses

Rugulies et al.	2020	Denmark	Cohort	Various<br><br>Most participants worked in public administration, education and health (41 and 42% in cohort I and II, respectively), followed by trade and transport (17% in both cohorts).	9981<br><br>53% women and 47% men	Major Depression Inventory (MDI)	Depressive symptom levels were statistically significantly higher among participants exposed to sexual harassment by both non-workplace personnel (Group 2, mean: 11.17, SD: 7.98, estimate (B): 1.61, 95% CI: 0.51 to 2.72, p = 0.004) and workplace personnel (Group 3, mean: 14.49, SD: 9.97, B: 3.85, 95% CI: 2.51 to 5.20, p < 0.001) compared to participants that were not exposed (Group 1, mean: 7.64, SD: 7.25) after adjustment for all covariates.<br><br>Compared to the reference group with no exposure to sexual harassment (Group 1), the odds ratio for incident depressive disorder was 1.92 (95% CI: 0.88 to 4.19, p = 0.10) among participants exposed to sexual harassment by non-workplace personnel (Group 2), and 5.26 (95% CI: 2.68 to 10.31, p < 0.001) among participants exposed to sexual harassment by workplace personnel (Group 3) after adjustment for all covariates.<br><br>From t1 to t3, depressive symptom levels remained stable in the non-exposed group (Group 1, +0.04 points) and increased for participants

							who became exposed to sexual harassment by non-workplace personnel (Group 2, +0.24 points) and workplace personnel (Group 3, +1.80 points).
Sumner et al.	2021	United States	Cohort	Military	502199<br><br>100% women	Depression diagnosis	Prevalence: 44.1% (depression) if screened positive for military sexual trauma (MST)<br><br>A positive MST screen was associated with an increased odds of receiving a diagnosis of depression: OR 2.81, 99% CI (2.76-2.88)<br><br>Results were similar, albeit attenuated, in the fully adjusted compared with age-adjusted models: OR 2.35, 99% CI (2.28-2.40)
Wu et al.	2016	Various	Cross-sectional	Commercial air pilots	1837<br><br>86.3% men and 13.7% women	Patient Health Questionnaire (PHQ-9)	Depression: 11.4% (experienced SHWP 1 time), 31.4% (2-3 times), 36.4% (4-more times)~~, 13.6% (total)~~
Yoo et al.	2018	South Korea	Cross-sectional	Health professionals<br><br>Emergency medical service (EMS)	1346<br><br>81.2% men and 18.8% women	The 11-item version of the Centers for Epidemiologic Studies Depression Scale (CES-D)	EMS providers who had experienced sexual harassment over the preceding year reported higher prevalence of current depressive symptoms: 49.3% in female and 37.7% in male who experienced SHWP vs. 18.1% and 19% who did not experience it.

							After controlling for potential covariates, the association between sexual harassment and depressive symptoms was significant in both women (PR: 2.97, 95% CI: 1.99, 4.44) and men (PR: 2.01, 95% CI: 1.41, 2.86).
Zhu et al.	2019	China	Cross-sectional	Hospitality workers	266<br><br>79.7% women and 20.3% men	Center for Epidemiological Studies Depression Scale (CESD)	SHWP was positively correlated with depression (r = 0.24, p < 0.01)
