# Supplementary material for: Systematic Review of Policies and Interventions to Prevent Sexual Harassment in the Workplace in Order to Prevent Depression
Source: Int J Environ Res Public Health. 2022 Oct 14;19(20):13278. doi: 10.3390/ijerph192013278 (PMC9603480; doi:10.3390/ijerph192013278)
Supplement: Supplementary file 1 [file ijerph-19-13278-s001.zip › Supplementary Material C.pdf]

Supplementary Material C. Included articles in review 2

Author(s)	Publication year	Country	Study design	Focus	Population	Sample size	Outcomes measured	Results
Antecol H, Cobb-Clark D	2003	United States	Cross-sectional	SHWP Training	Civil employees of the US federal government	5875<br><br>Gender distribution not specified	Label unwanted sexual behaviors as SHWP<br>Experience with training<br>Perception of impact of training on their beliefs<br>Perception of training on SHWP prevention in their organization	Half of respondents reported training made them more sensitive to the issue.<br><br>Training is associated with increases in the probability that employees label behaviors as forms of sexual harassment. The effect was higher on men than women.
Buckner et al.	2014	United States	Cross-sectional	SHWP Training	Managers	209<br><br>62% men and 38% women	Sexual harassment identification and appropriate response<br>Prior Training	Quantity of training and the interaction between quantity and recency predicted identification sensitivity.<br><br>None of the training variables predicted identification accuracy.
Campbell et al.	2013	United States	Quasi-experimental	SHWP Training	Employees from three workplaces (service, military and social service)	88 total<br><br>52% women and 38% men<br><br>44 in intervention group<br>36 in control group	Knowledge of SHWP prevention	Significant improvement in SHWP knowledge between pretest and posttest.<br><br>Significant differences in knowledge between intervention and control group.

De Haas et al.	2010	Netherlands	Cross-sectional	Policy	Police	6387 in 2000<br>53% men and 47% women<br><br>4296 in 2006<br>70% men and 30% women	Experience with SHWP	Eight divisions had comprehensive policies (written policy statement, preventive efforts aiming to change the organizational climate, a grievance procedure, information and training, and confidential advisors) and eight had fewer comprehensive policies.<br><br>Similar percentages of men and women with experiences of sexual harassment were found in divisions with comprehensive (67% for women, 49% for men) and less comprehensive policies (62% for women, 48% for men)
Dobbin F, Kalev A	2019	United States	Cross-sectional	Policy	Managers	805 companies	SHWP programs, practices and policies<br>Gender, race and ethnic composition of workplaces	Grievance procedures hinder access of women to management positions where men dominate.<br><br>This effect disappears where women hold more management jobs.<br><br>SHWP training is followed by an increase of women of color in management.

Estrada A, Laurence J	2009	United States	Cross-sectional	SHWP Training	Military (Army, Navy and Air Force)	71570<br><br>83.8% men and 16.2% women	Experience with policy training<br>Perception of training effectiveness<br>Understanding of policy<br>Harassment intolerance	Men and women who received training rated the policy as more effective than those who did not receive it.<br><br>Men and women trained scored higher on knowledge and understanding of the policy<br><br>Men and women who received training reported higher levels of intolerance to harassment.
Fawole et al.	2005	Nigeria	Quasi-experimental	SHWP Training	Young apprentices	323 women for intervention<br><br>203 women for follow-up	Knowledge of physical and sexual violence and consequences<br>Prevalence of different forms of violence	SHWP knowledge significantly increased after training.<br><br>Prevalence of physical violence was reduced at follow-up (65% to 23%).
Glass et al.	2017	United States	Quasi-experimental	SHWP Training<br><br>Computer-based + peer group versus computer-based only	Homecare workers	306 women total<br><br>152 in computer-based training + peer group<br>154 in computer-based training only group	Identification of violence and harassment<br>Confidence in prevention SHWP<br>Stress, depression and sleep (COPSOQ II)<br>Burnout (Copenhagen Burnout Inventory)	Knowledge significantly increased in both groups' posttest compared to pretest.<br><br>No significant differences were found in change over time in confidence to respond to SHWP.<br><br>All participants reported a decrease in SHWP over time.

								No significant difference in stress, depression, sleep or burnout between groups
Goldberg C	2007	United States	RCT	SHWP Training	White-collar professionals from a variety of industries	234<br><br>55% male and 45% female	Intended responses to SHWP<br>Conflict avoidance	Participants in the training condition expressed a lower likelihood of confronting the perpetrator than did control group subjects.<br><br>No significant relationship between conflict avoidance and intention to confront the perpetrator.<br><br>Significant negative relationship between conflict avoidance and intention to report.
Hock et al.	2021	Not specified	Quasi-experimental	SHWP Training	Physicians	91<br><br>50% female, 48% male, 2% not answered	Preparedness to respond to SHWP<br>Previous experience with SHWP<br>Experience with training	Following training, there was a significant improvement in preparedness, particularly to milder forms of SHWP.<br><br>Men rated themselves as more prepared before the training. After the training this rating remained but there were no significant differences with women.

Jacobson RK, Eaton AA	2017	United States	RCT	Policy	Employees of a Human Resource Department at a large public university	101<br><br>74.26% women and 25.74% men	Likelihood of reporting SHWP<br>Identification of SHWP	Those assigned to the zero-tolerance condition were significantly more likely to report the behavior compared to standard policy an no policy conditions.<br><br>The effect of the policy on reporting was stronger in the moderate harassment scenario than the severe harassment scenario.<br><br>Those who read the severe harassment scenario were more likely to report than those who read the moderate scenario.
Perry et al.	2010	United States	Cross-sectional	SHWP Training	Human Resource workers	321<br><br>Gender distribution not specified	Perceived training effectiveness<br>Number of pretraining and post training activities<br>Number of active and passive training methods used<br>Perceived reason for training	Model revealed no significant effects of number of pretraining activities, number of active or passive training methods, or number of post training activities on perceived training success.<br><br>The number of pretraining and post training activities, had a significant and positive effect on perceived effectiveness when the reason was strategic.<br><br>The more post training activities, the lower the

								perceived frequency of sexual harassment complaints.
Preusser et al.	2011	United States	RCT	SHWP Training<br><br>Computer-based versus instructor-led	University employees	70<br><br>49% men and 51% women	Training type preference<br>Cognitive, skill-based and affective learning<br>Reactions to the training	Scores on all learning measures increased in both groups.<br><br>Participant reactions were positive regardless of type of training delivered.<br><br>No significant differences between instructor-led and computer-based training on cognitive or skill-based learning.
Relyea et al.	2020	United States	Cross-sectional	SHWP Training	Veterans' Health Administration Staff	180<br><br>65.6% women, 20% men, 14.4% unknown	Experience of SHWP<br>Experiences with witnessing or disclosure of SHWP<br>History of intervening to stop SHWP<br>Awareness of SHWP<br>Barriers to intervening<br>Self-efficacy<br>Intention to intervene<br>Training acceptability	Comparing pretest and posttest, there were a significant decrease in perceived barriers to intervening, and an increase in self-efficacy and intentions to intervene. Effect sizes were small.<br><br>Perceived awareness of SHWP increased from 42.24% to 75%.<br><br>Opposite effects were found in 7.7% regarding barriers, 2.7% regarding self-efficacy and 6.1% regarding intentions to intervene. These

								participants reported during pretest not believing SHWP was a problem in their workplace.
Ridenour et al.	2017	United States	Cross-sectional	Policy	Registered and licensed practical nurses	309<br><br>90% women, 10% men	Knowledge of the NJ violence prevention in health care facilities act<br>Training received<br>Experience of work-related violence	A higher proportion of respondents who heard of the regulation experienced sexual harassment compared to those who had not heard about it.<br><br>Respondents heard of the act had received a higher proportion of training than those who did not.<br><br>Respondents who received training of the required components were more likely to be RNs, had heard of the NJ act, and had less tenure.
Shapiro et al.	2014	United States	Cross-sectional	Policy	Health professionals	201<br><br>Gender distribution not specified	Perception of effectiveness<br>Perception of improved understanding of professionalism<br>Perception of benefits to their professional practice	The number of reports has steadily increased each year, with 45, 51, and 71 submitted in 2010, 2011, and 2012, respectively.<br><br>The most common intervention was a feedback conversation with the focus person (FP) and his or her supervisory physician, usually facilitated by the CPPS

	director or associate director. Some FPs underwent behavioral coaching.<br><br>Some FPs, although initially angered or confused at being identified as unprofessional, were subsequently appreciative of the process.
