# Supplementary material for: Systematic Review of Policies and Interventions to Prevent Sexual Harassment in the Workplace in Order to Prevent Depression
Source: Int J Environ Res Public Health. 2022 Oct 14;19(20):13278. doi: 10.3390/ijerph192013278 (PMC9603480; doi:10.3390/ijerph192013278)
Supplement: Supplementary file 1 [file ijerph-19-13278-s001.zip › Supplementary Material D.pdf]

**Supplementary Material D. Quality assessment of studies in review 1**

Tool used: Appraisal tool for Cross-Sectional Studies (AXIS tool) / *NR: Not reported

| | Abo | Celik | Dutra | Fang | Friborg | Gale | Gross | Hanson | Hom | Houle | Kim | Malik | Marsh | Mathisen | Matud | McCallum | Millegan2 | Murdoch | Mushtaq | Rugulies | Sumner | Wu | Yoo | Zhu |
|---|---|---|---|---|---|---|---|---|---|---|---|---|---|---|---|---|---|---|---|---|---|---|---|---|
| ***Introduction*** | | | | | | | | | | | | | | | | | | | | | | | | |
| 1 Were the aims/objectives of the study clear? | Yes | Yes | Yes | Yes | Yes | Yes | Yes | Yes | Yes | Yes | Yes | Yes | Yes | Yes | Yes | Yes | Yes | Yes | Yes | Yes | Yes | Yes | Yes | Yes |
| ***Methods*** | | | | | | | | | | | | | | | | | | | | | | | | |
| 2 Was the study design appropriate for the stated aim(s)? | Yes | Yes | Yes | Yes | Yes | Yes | Yes | Yes | Yes | Yes | Yes | Yes | Yes | Yes | Yes | Yes | Yes | Yes | Yes | Yes | Yes | Yes | Yes | Yes |
| 3 Was the sample size justified? | Yes | No | No | Yes | Yes | Yes | Yes | Yes | No | No | Yes | No | No | No | No | No | No | No | No | No | No | No | No | No |
| 4 Was the target/reference population clearly defined? (Is it clear who the research was about?) | Yes | Yes | Yes | Yes | Yes | Yes | Yes | Yes | Yes | Yes | Yes | Yes | Yes | Yes | Yes | Yes | Yes | Yes | Yes | Yes | Yes | Yes | Yes | Yes |
| 5 Was the sample frame taken from an appropriate population base so that it closely represented the target/reference population under investigation? | Yes | Yes | Yes | Yes | Yes | Yes | Yes | Yes | Yes | Yes | Yes | Yes | Yes | Yes | Yes | Yes | Yes | Yes | Yes | Yes | Yes | Yes | Yes | Yes |
| 6 Was the selection process likely to select subjects/participants that were representative of the target/reference population under investigation? | Yes | Yes | Yes | Yes | Yes | Yes | Yes | Yes | Yes | Yes | Yes | Yes | Yes | Yes | Yes | Yes | Yes | Yes | Yes | Yes | Yes | Yes | Yes | Yes |
| 7 Were measures undertaken to address and categorise non-responders? | Yes | No | NR | NR | NR | NR | Yes | Yes | NR | NR | NR | NR | No | NR | NR | NR | NR | NR | NR | NR | NR | NR | NR | NR |
| 8 Were the risk factor and outcome variables measured appropriate to the aims of the study? | Yes | Yes | Yes | Yes | NR | Yes | Yes | Yes | Yes | Yes | Yes | Yes | Yes | Yes | Yes | Yes | Yes | Yes | Yes | Yes | Yes | Yes | Yes | Yes |
| 9 Were the risk factor and outcome variables measured correctly using instruments/ measurements that had been trialled, piloted or published previously? | No | Yes | Yes | Yes | NR | Yes | Yes | Yes | Yes | Yes | Yes | No | Yes | Yes | Yes | Yes | Yes | Yes | Yes | Yes | Yes | Yes | Yes | Yes |
| 10 Is it clear what was used to determined statistical significance and/or precision estimates? (eg, p values, CIs) | Yes | Yes | Yes | Yes | Yes | Yes | Yes | Yes | Yes | Yes | Yes | Yes | Yes | Yes | Yes | Yes | Yes | Yes | No | Yes | Yes | Yes | Yes | No |
| 11 Were the methods (including statistical methods) sufficiently described to enable them to be repeated? | No | Yes | Yes | Yes | Yes | Yes | Yes | Yes | Yes | Yes | No | Yes | Yes | Yes | No | Yes | Yes | Yes | No | Yes | Yes | Yes | Yes | No |
| ***Results*** | | | | | | | | | | | | | | | | | | | | | | | | |
| 12 Were the basic data adequately described? | Yes | Yes | Yes | Yes | Yes | Yes | Yes | Yes | Yes | Yes | Yes | Yes | Yes | Yes | Yes | No | Yes | Yes | Yes | Yes | Yes | Yes | No | No |
| 13 Does the response rate raise concerns about non-response bias? | Yes | No | No | NR | NR | NR | No | No | NR | NR | No | No | No | No | No | No | No | No | No | No | No | No | No | No |
| 14 If appropriate, was information about non-responders described? | No | No | No | NR | NR | NR | No | No | No | No | No | No | No | No | No | Yes | No | No | No | No | No | No | No | No |
| 15 Were the results internally consistent? | NR | NR | NR | NR | NR | Yes | Yes | NR | Yes | NR | NR | NR | Yes | Yes | NR | NR | NR | Yes | Yes | NR | Yes | NR | NR | Yes |
| 16 Were the results for the analyses described in the methods, presented? | Yes | Yes | Yes | Yes | Yes | Yes | Yes | Yes | Yes | Yes | Yes | Yes | Yes | Yes | No | Yes | Yes | Yes | Yes | Yes | Yes | Yes | Yes | Yes |
| ***Discussion*** | | | | | | | | | | | | | | | | | | | | | | | | |
| 17 Were the authors' discussions and conclusions justified by the results? | Yes | Yes | Yes | Yes | Yes | Yes | Yes | Yes | Yes | Yes | Yes | Yes | Yes | Yes | Yes | Yes | Yes | Yes | Yes | Yes | Yes | Yes | Yes | Yes |
| 18 Were the limitations of the study discussed? | Yes | No | Yes | Yes | Yes | Yes | Yes | Yes | Yes | No | No | Yes | No | Yes | Yes | Yes | Yes | No | Yes | Yes | Yes | Yes | Yes | Yes |
| ***Other*** | | | | | | | | | | | | | | | | | | | | | | | | |
| 19 Were there any funding sources or conflicts of interest that may affect the authors' interpretation of the results? | No | NR | NR | NR | No | No | No | No | No | NR | NR | No | NR | No | NR | No | NR | NR | NR | No | NR | No | No | NR |
| 20 Was ethical approval or consent of participants attained? | Yes | Yes | Yes | Yes | Yes | Yes | Yes | Yes | Yes | Yes | Yes | Yes | NR | Yes | Yes | Yes | Yes | Yes | Yes | NR | Yes | Yes | Yes | NR |
