# Supplementary material for: Systematic Review of Policies and Interventions to Prevent Sexual Harassment in the Workplace in Order to Prevent Depression
Source: Int J Environ Res Public Health. 2022 Oct 14;19(20):13278. doi: 10.3390/ijerph192013278 (PMC9603480; doi:10.3390/ijerph192013278)
Supplement: Supplementary file 1 [file ijerph-19-13278-s001.zip › Supplementary Material E.pdf]

Supplementary Material E. Quality assessment of studies in review 2

	A) SELECTION BIAS			B) STUDY DESIGN					C) CONFOUNDERS			D) BLINDING		
	**(Q1) Are the individuals selected to participate in the study likely to be representative of the target population?**<br>1 Very likely<br>2 Somewhat likely<br>3 Not likely<br>4 Can't tell	**(Q2) What percentage of selected individuals agreed to participate?**<br>1 80 - 100% agreement<br>2 60 – 79% agreement<br>3 less than 60% agreement<br>4 Not applicable<br>5 Can't tell	**RATE THIS SECTION**<br>1 STRONG<br>2 MODERATE<br>3 WEAK	**Indicate the study design**<br>1 Randomized controlled trial<br>2 Controlled clinical trial<br>3 Cohort analytic (two group pre + post)<br>4 Case-control<br>5 Cohort (one group pre + post (before and after))<br>6 Interrupted time series<br>7 Other (specify)<br>8 Can't tell	**Was the study described as randomized?**<br>***If NO, go to Component C.***<br>No<br>Yes	**If Yes, was the method of randomization described?**<br>No<br>Yes	**If Yes, was the method appropriate?**<br>No<br>Yes	**RATE THIS SECTION**<br>1 STRONG<br>2 MODERATE<br>3 WEAK	**(Q1) Were there important differences between groups prior to the intervention?**<br>1 Yes<br>2 No<br>3 Can't tell	**(Q2) If yes, indicate the percentage of relevant confounders that were controlled (either in the design (e.g. stratification, matching) or analysis)?**<br>1 80 – 100% (most)<br>2 60 – 79% (some)<br>3 Less than 60% (few or none)<br>4 Can't Tell	**RATE THIS SECTION**<br>1 STRONG<br>2 MODERATE<br>3 WEAK	**(Q1) Was (were) the outcome assessor(s) aware of the intervention or exposure status of participants?**<br>1 Yes<br>2 No<br>3 Can't tell	**(Q2) Were the study participants aware of the research question?**<br>1 Yes<br>2 No<br>3 Can't tell	**RATE THIS SECTION**<br>1 STRONG<br>2 MODERATE<br>3 WEAK
Antecol	1	4	3	7 (cross-sectional)	No	N/A	N/A	3	3	N/A	3	N/A	3	3
Buckner	3	5	3	7 (cross-sectional)	No	N/A	N/A	3	3	N/A	3	N/A	3	3
Campbell	3	3	3	3	No	N/A	N/A	2	3	N/A	3	2	3	2
De Haas	2	3	3	7 (cross-sectional)	No	N/A	N/A	3	3	N/A	3	N/A	3	3
Dobbin	1	5	3	7 (cross-sectional)	No	N/A	N/A	3	3	N/A	3	N/A	3	3
Estrada	1	5	3	7 (cross-sectional)	No	N/A	N/A	3	3	N/A	3	N/A	3	3
Fawole	2	5	3	5	No	N/A	N/A	2	3	N/A	3	2	3	2
Glass	3	5	3	3	Yes	Yes	Yes	2	2	N/A	1	2	3	2
Goldberg	3	5	3	1	Yes	No	N/A	1	2	N/A	1	2	3	2
Hock	3	5	3	5	No	N/A	N/A	3	3	N/A	3	2	3	2
Jacobson	3	3	3	1	Yes	No	N/A	2	3	N/A	3	2	2	1
Perry	2	5	3	7 (cross-sectional)	No	N/A	N/A	3	3	N/A	3	2	3	2
Preusser	2	5	3	3	Yes	No	N/A	2	3	N/A	3	2	3	2
Relyea	3	5	3	7 (cross-sectional)	No	N/A	N/A	3	3	N/A	3	2	3	2
Ridenour	1	3	3	7 (cross-sectional)	No	N/A	N/A	3	3	N/A	3	2	3	2
Shapiro	3	5	3	7 (cross-sectional)	No	N/A	N/A	3	3	N/A	3	1	3	2

	E) DATA COLLECTION METHODS			F) WITHDRAWALS AND DROP-OUTS			G) INTERVENTION INTEGRITY			H) ANALYSES				GLOBAL RATING
	**(Q1) Were data collection tools shown to be valid?** 1 Yes 2 No 3 Can't tell	**(Q2) Were data collection tools shown to be reliable?** 1 Yes 2 No 3 Can't tell	**RATE THIS SECTION** 1 STRONG 2 MODERATE 3 WEAK	**(Q1) Were withdrawals and drop-outs reported in terms of numbers and/or reasons per group?** 1 Yes 2 No 3 Can't tell 4 Not Applicable (i.e. one time surveys or interviews)	**(Q2) Indicate the percentage of participants completing the study.** ***(If the percentage differs by groups, record the lowest)*.** 1 80 -100% 2 60 - 79% 3 less than 60% 4 Can't tell 5 Not Applicable (i.e. Retrospective case-control)	**RATE THIS SECTION** 1 STRONG 2 MODERATE 3 WEAK 4 NOT APPLICABLE	**(Q1) What percentage of participants received the allocated intervention or exposure of interest?** 1 80 -100% 2 60 - 79% 3 less than 60% 4 Can't tell	**(Q2) Was the consistency of the intervention measured?** 1 Yes 2 No 3 Can't tell	**(Q3) Is it likely that subjects received an unintended intervention (contamination or co-intervention) that may influence the results?** 1 Yes 2 No 3 Can't tell	**(Q1) Indicate the unit of allocation** ***(choose only one)*** community organization/institution practice/office individual	**(Q2) Indicate the unit of analysis** ***(choose only one)*** community organization/institution practice/office individual	**(Q3) Are the statistical methods appropriate for the study design?** 1 Yes 2 No 3 Can't tell	**(Q4) Is the analysis performed by intervention allocation status (i.e. intention to treat) rather than the actual intervention received?** 1 Yes 2 No 3 Can't tell	**GLOBAL RATING FOR THIS PAPER** ***(choose only one)* :** 1 STRONG *(no WEAK ratings)* 2 MODERATE *(one WEAK rating)* 3 WEAK *(two or more WEAK ratings)*
Antecol	1	1	1	4	5	4	N/A	N/A	N/A	N/A	N/A	N/A	N/A	3
Buckner	1	1	1	4	5	4	N/A	N/A	N/A	N/A	N/A	N/A	N/A	3
Campbell	1	1	1	2	1	1	1	3	2	Individual	Individual	1	2	3
De Haas	1	1	1	4	5	4	N/A	N/A	N/A	N/A	N/A	N/A	N/A	3
Dobbin	1	1	1	4	5	4	N/A	N/A	N/A	N/A	N/A	N/A	N/A	3
Estrada	1	1	1	4	5	4	N/A	N/A	N/A	N/A	N/A	N/A	N/A	3
Fawole	1	1	1	1	4	3	1	3	2	Individual	Individual	1	2	3
Glass	1	1	1	1	1	1	1	1	2	Individual	Individual	1	2	2
Goldberg	1	1	1	2	4	3	1	3	2	Individual	Individual	1	2	2
Hock	1	1	1	2	4	3	1	3	2	Individual	Individual	1	2	3
Jacobson	1	1	1	2	1	1	1	3	2	Individual	Individual	1	2	3
Perry	1	1	1	4	5	4	N/A	N/A	N/A	N/A	N/A	N/A	N/A	3
Preusser	1	1	1	2	4	3	1	3	2	Individual	Individual	1	2	3
Relyea	1	1	1	3	5	4	N/A	N/A	N/A	N/A	N/A	N/A	N/A	3
Ridenour	1	1	1	4	5	4	N/A	N/A	N/A	N/A	N/A	N/A	N/A	3
Shapiro	1	1	1	4	5	4	N/A	N/A	N/A	N/A	N/A	N/A	N/A	3

Tool used: Effective Public Health Practice Project Quality Assessment Tool for Quantitative Studies (EPHPP)
